# Repeated Single-lung Transplantation After Previous Contralateral Pneumonectomy—A Unique Challenge

**DOI:** 10.1097/TXD.0000000000001613

**Published:** 2024-03-12

**Authors:** Birte Ohm, Johannes Kalbhenn, Ina Hettich, Florian Emmerich, Axel Semmelmann, Johannes Ortmann, Martin Czerny, Daiana Stolz, Wolfgang Jungraithmayr

**Affiliations:** 1 Department of Thoracic Surgery, Medical Center–University of Freiburg, Faculty of Medicine, University of Freiburg, Germany.; 2 Department of Anesthesiology and Critical Care, Medical Center–University of Freiburg, Faculty of Medicine, University of Freiburg, Germany.; 3 Department of Pneumology, Medical Center–University of Freiburg, Faculty of Medicine, University of Freiburg, Germany.; 4 Institute for Transfusion Medicine and Gene Therapy, Medical Center–University of Freiburg, Faculty of Medicine, University of Freiburg, Germany.; 5 Department of Cardiovascular Surgery, Faculty of Medicine, University Heart Center Freiburg, Bad Krozingen, University of Freiburg, Germany.; 6 Lungenklinik Breisgau, Waldkirch, Germany.

Unilateral lung transplantation after previous contralateral pneumonectomy is a rare clinical situation and is associated with an increased risk of perioperative morbidity and mortality. Such patients, reliant on their single transplant lung, are substantially threatened by graft failure.

We here provide the first report of a successful single-lung retransplantation for chronic lung allograft dysfunction (CLAD) after previous contralateral pneumonectomy.

## CASE REPORT

A 37-y-old female patient initially presented with severe bronchiectasis complicated by atypical mycobacteriosis. She received antimycobacterial therapy and was listed for lung transplantation. Due to progressive destruction of the left lung, the patient underwent a left-sided pneumonectomy. Shortly thereafter, she received an organ for right-sided single-lung transplantation. The postoperative course was uneventful except for prolonged weaning, and the patient performed well for the following 8 y. Pulmonary function testing showed a postoperative baseline forced expiratory volume in 1 s of 1.51 L.

**FIGURE 1.**
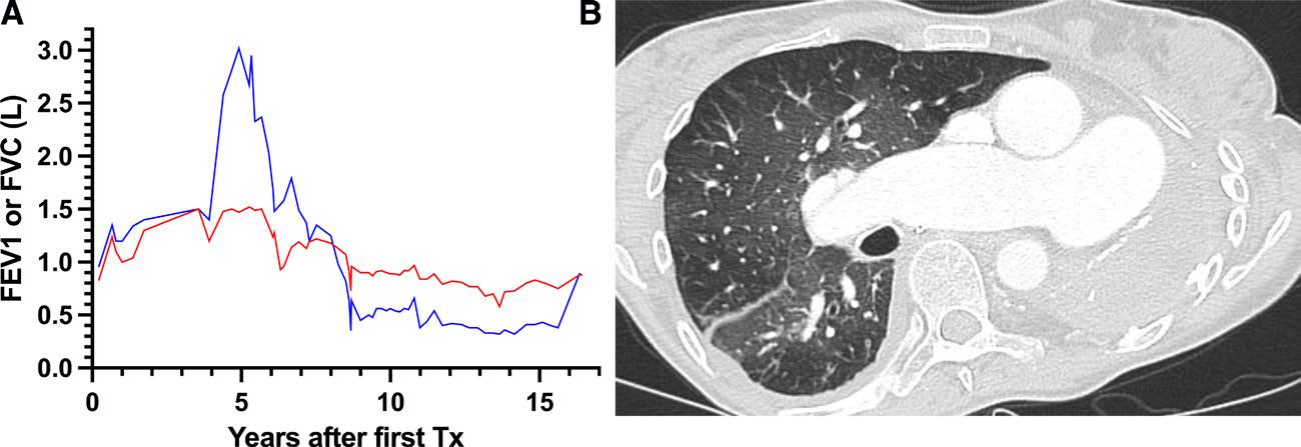
Patient characteristics before re-Tx. The patient experienced a decline of pulmonary function (blue line = FVC, red line = FEV_1_) because of a mixed phenotype of CLAD (A). Computed tomography before retransplantation demonstrates CLAD-related opacities, a vastly changed anatomy because of contralateral pneumonectomy, and an enlarged diameter of the pulmonary artery due to pulmonary hypertension (B). CLAD, chronic lung allograft dysfunction; FEV_1_, forced expiratory volume in 1 s; FVC, forced vital capacity; Tx, transplantation.

Eight years after transplantation (Figure 1A), the patient’s pulmonary function gradually declined, evidenced by progressive obstruction and restriction. Computed tomography revealed parenchymal opacities within the transplanted lung (Figure 1B), confirming a definite diagnosis of a mixed phenotype of CLAD.

At that time, a right-sided single-lung retransplantation was recommended. However, the patient initially refused retransplantation for personal reasons. Eventually, 15 y after her first transplantation, she agreed to a retransplant procedure because of further worsening of the respiratory performance reflected by progressive hypercapnia. Right heart catheterization revealed newly developed precapillary pulmonary hypertension (39/26 mm Hg). At retransplantation, the patient was aged 54 y and presented with a height of 175 cm and a body weight of 47 kg (body mass index 15.3). The organ was procured from a 12-y-old female donor who had anoxic brain damage during resuscitation because of drug intoxication with a cardioactive substance. Having a height of 162 cm and a body weight of 52 kg, donor organ size matched the recipient despite her young age.

After the establishment of a femorofemoral cardiopulmonary bypass, the implantation procedure was uneventful. Extracorporeal circulation ran for 115 min and was successfully terminated shortly after organ reperfusion.

**FIGURE 2.**
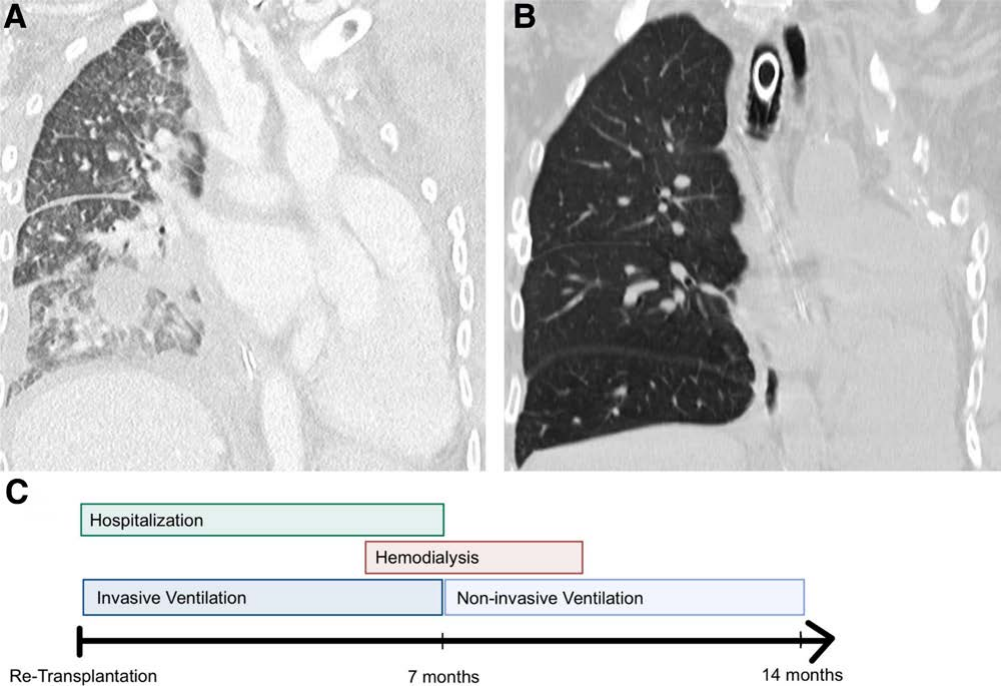
Computed tomography revealed interstitial and alveolar edema at 1 mo postoperatively (A). All infiltrates had completely resolved when the patient was discharged for rehabilitation several months later (B). The postoperative clinical course was prolonged because of the necessity of hemodialysis and invasive ventilation (C).

The postoperative course was characterized by protracted cardiorespiratory adaptation requiring catecholamine therapy for 2 mo. Due to prior pneumonectomy and vascular remodeling during CLAD development, the patient had developed right ventricular hypertrophy. Postoperatively, the reduction of the pulmonary artery pressure within the graft caused a hyperdynamic circulatory state with hypervolemic pulmonary circulation. Progressive right ventricular dysfunction was evidenced by tricuspid insufficiency on echocardiography along with pulmonary edema (Figure 2A). The patient developed cardiorenal syndrome and required renal replacement therapy. The hemodynamic condition continuously improved by managing negative fluid balance, beta-adrenergic blockade, and the reduction of afterload by prostaglandin treatment. With regard to the respiratory state, the patient initially had ventilatory insufficiency because of pulmonary cachexia and weakness of the respiratory muscles. As a prolonged weaning period was anticipated, she underwent dilatative tracheostomy directly after retransplantation. Seven months after transplantation, she was successfully decannulated. Today, 14 mo after retransplantation, the patient relies only on noninvasive ventilatory support at night. During daytime, she is free from any oxygen supplementation at rest. The circulatory situation fully recovered without any evidence of right heart failure. The postoperative course is summarized in Figure 2C.

## DISCUSSION

As no effective treatment is available against CLAD, lung retransplantation is the only definite treatment option. However, survival after retransplantation is inferior to primary transplantation and candidate selection for a retransplantation strongly relies on interdisciplinary decision-making.^1^ The case presented here is to our knowledge the first description of a successful retransplantation for CLAD in a single-lung-transplanted patient after previous pneumonectomy. The case should demonstrate which therapeutic measures might deteriorate or ameliorate the postoperative course in light of an atypical pathophysiological situation.

Unilateral lung transplantation after previous pneumonectomy has been associated with a higher risk for perioperative morbidity and mortality because of anatomical alterations by mediastinal shift.^2,3^ Contrary to these reports, a recent study by the European Society of Thoracic Surgery Lung Transplantation Working Group claims that any history of anatomic lung resection, including pneumonectomy, results in similar outcomes to conventional lung transplantation.^4^ However, the authors here described a small population of patients and included only 2 cases of previous contralateral pneumonectomy.

Pneumonectomy results in long-term hemodynamic changes. Right ventricular hypertrophy and tricuspid valve insufficiency are often evident for several years after pneumonectomy.^5^ A mildly increased pre-retransplant pulmonary hypertension, as seen in our patient, can aggravate the clinical course. Shortly after transplantation, pulmonary vascular resistance decreased, resulting in volume overload and dilation of the left ventricle. Consequently, this led to pulmonary edema. Renal replacement therapy eventually resolved this issue. However, to prevent such complications, it might have been beneficial to delay the removal of extracorporeal support shortly after transplantation. By prolonging support with the venoarterial extracorporeal membrane oxygenation, it could be postulated that right heart preload would have been reduced, thus protecting the patient’s end organs, particularly the kidneys. Also, this approach would have potentially reduced the need for vasopressors and ventilatory support over time. Consequently, it could have allowed for right ventricular remodeling and left ventricular diastolic accommodation.^6^

In summary, unilateral retransplantation of a primarily pneumonectomized patient can be a justified option in selected cases. However, in anticipation of severe pathophysiological obstacles, the prolonged use of venoarterial extracorporeal membrane oxygenation beyond the immediate postoperative phase might be advantageous to facilitate ventricular remodeling and thus protect both remote organs and the transplanted lung.
